# Intracellular Diagnostics: Hunting for the Mode of Action of Redox-Modulating Selenium Compounds in Selected Model Systems

**DOI:** 10.3390/molecules190812258

**Published:** 2014-08-13

**Authors:** Dominika Mániková, Lucia Medvecová Letavayová, Danuša Vlasáková, Pavol Košík, Ethiene Castellucci Estevam, Muhammad Jawad Nasim, Martin Gruhlke, Alan Slusarenko, Torsten Burkholz, Claus Jacob, Miroslav Chovanec

**Affiliations:** 1 Department of Genetics, Cancer Research Institute, Vlárska 7, 833 91 Bratislava, Slovakia; E-Mails: dominika.manikova@savba.sk (D.M.); lucia.letavayova@gmail.com (L.M.L.); exondako@savba.sk (D.V.); 2 Department of Oncology, Cancer Research Institute, Vlárska 7, 833 91 Bratislava, Slovakia; E-Mail: pavol.kosik@savba.sk; 3 Division of Bioorganic Chemistry, School of Pharmacy, Saarland University, Campus B 2.1, D-66123 Saarbruecken, Germany; E-Mails: ethiene.castellucciestevam@uni-saarland.de (E.C.E.); jawad.nasim@uni-saarland.de (M.J.N.); t.burkholz@mx.uni-saarland.de (T.B.); 4 CAPES Foundation, Ministry of Education of Brazil, DF 70040-020 Brasilia, Brazil; 5 Department of Plant Physiology (BioIII), RWTH Aachen University, D-52056 Aachen, Germany; E-Mails: Martin.Gruhlke@rwth-aachen.de (M.G.); alan.slusarenko@bio3.rwth-aachen.de (A.S.)

**Keywords:** chemogenomic screening, intracellular thiolstat, intracellular diagnostics, redox modulation, nematodes, *Saccharomyces cerevisiae*, thiolstat

## Abstract

Redox-modulating compounds derived from natural sources, such as redox active secondary metabolites, are currently of considerable interest in the field of chemoprevention, drug and phytoprotectant development. Unfortunately, the exact and occasionally even selective activity of such products, and the underlying (bio-)chemical causes thereof, are often only poorly understood. A combination of the nematode- and yeast-based assays provides a powerful platform to investigate a possible biological activity of a new compound and also to explore the “redox link” which may exist between its activity on the one side and its chemistry on the other. Here, we will demonstrate the usefulness of this platform for screening several selenium and tellurium compounds for their activity and action. We will also show how the nematode-based assay can be used to obtain information on compound uptake and distribution inside a multicellular organism, whilst the yeast-based system can be employed to explore possible intracellular mechanisms via chemogenetic screening and intracellular diagnostics. Whilst none of these simple and easy-to-use assays can ultimately substitute for in-depth studies in human cells and animals, these methods nonetheless provide a first glimpse on the possible biological activities of new compounds and offer direction for more complicated future investigations. They may also uncover some rather unpleasant biochemical actions of certain compounds, such as the ability of the trace element supplement selenite to induce DNA strand breaks.

## 1. Introduction

The last decade has witnessed a rapidly growing interest in redox modulating natural products, from traditional antioxidants to more sophisticated modulatory agents which may affect not only individual target proteins, but also entire signaling pathways. Some of these substances may even cause epigenetic changes, for instance by chemically modifying histones. Indeed, redox active natural products bear considerable promise as antioxidants (e.g., in the preservation of food), as essential ingredients of our daily food and also as nutritional supplements (e.g., chemopreventive agents). Some of these compounds, such as allicin from garlic and sulforaphane from broccoli are even being considered in the context of drug development and as potent phytoprotectants (e.g., “green pesticides”). Not surprisingly, a whole barrage of new and chemically diverse redox active secondary metabolites has recently emerged. As this “Special Issue” illustrates nicely, many of these products are currently considered in the context of health, medicine and agriculture.

It is likely that the number of new redox active natural products, isolated from ever more exotic plants from equally exotic places, is going to continue to grow at a breathtaking pace. At the same time, however, biology is faced with the rather urgent need to establish not only the “biological activity” of many of such new products, but also to investigate their (cellular) targets and likely mode(s) of action. Given the considerable time pressure caused by the emergence of ever more extracts, mixtures and ultimately chemically well-defined new compounds, this task is far from trivial.

**Figure 1:**
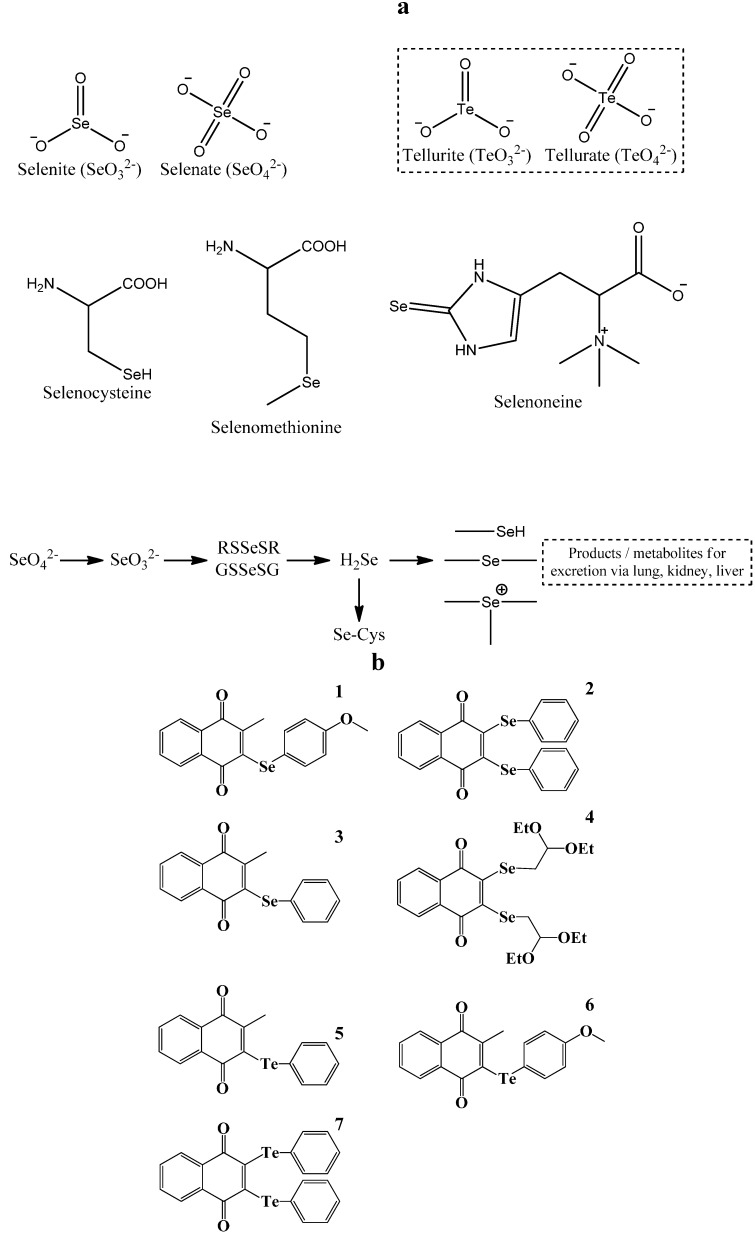
The biological “chemistry” of selenium and tellurium. **Panel a:** Selenium occurs in biological systems mostly as selenocysteine and selenomethionine, two amino acids which are found in various proteins and enzymes. Other organic forms of selenium are less abundant. They are often associated with selenium metabolism (e.g., dimethylselenide, glutathione selenotrisulfide) or resemble organic sulfur compounds (e.g., selenoneine); **Panel b:** Chemical structures of the various selenium and tellurium compounds employed as part of this study.

It has therefore been our aim to develop a “simple”, robust and reliable test system for biological activity which at the same time enables more in-depth and diversified studies if desired. To address this objective, we have departed from our usual and time-consuming (mammalian, human) cell culture studies and have explored avenues to establish the biological activity of compounds in a fast, effective and straightforward manner. At the same time, we have tried to develop a simple platform to study the “redox link” which ultimately may exist between the chemistry and biochemistry of a given compound on the one hand, and its biological activity on the other. For this task, we have chosen a rather simple, redox active natural “product”, namely selenium, a trace element which occurs naturally in various forms and can also be accessed readily by organic synthetic chemistry. Selenium is present in the soil primarily as selenite (SeO_3_^2−^) and selenate (SeO_4_^2−^). Most organisms, including plants, are able to metabolize these inorganic forms of selenium in order to synthesize a wide range of reactive organic selenium compounds (OSeCs). These compounds include better known metabolites, such as hydrogen selenide (H_2_Se), dimethylselenide ((CH_3_)_2_Se), the trimethylselenium ion ((CH_3_)_3_Se^+^), selenocysteine and selenomethionine, but also a few more “exotic” molecules, such as selenoneine, which has been discovered recently in rather impressive amounts in the blood of bluefin tuna [1]. A selection of selenium compounds found in Nature and the predominant metabolic pathways of selenium in (higher) organisms are shown in Figure 1, together with the chemical structures of the compounds used as part of this study.

Indeed, redox-active selenium is of utmost importance in human health and disease, and its dosage is often critical. A lack of selenium in the diet, which occurs for instance in certain regions of China, can have dramatic effects, ranging from malnutrition, retarded growth and impotence to more serious disorders, such as Keshan disease, Khasin-Beck disease and even increased incidences of cancer. Yet an excessive uptake of selenium is equally dangerous and selenium, when administered in high(er) amounts, seems to turn toxic. Not surprisingly, a whole field of research has emerged during the last two decades which is focused on selective cytotoxic properties of various selenium compounds. Such molecules may be used, for instance, to selectively target and kill cancer cells, to shut down or kill activated macrophages causing havoc in inflammatory diseases or to eliminate microbes (e.g., bacteria, plasmodia, nematodes) during infections [2,3,4,5,6].

Since the early 1990s, numerous publications have appeared covering aspects of redox modulation based on selenium and related chalcogen compounds [7,8], yet most of these studies have been rather tedious to conduct, and in most cases, the precise mechanism(s) responsible for this particular cytotoxic action are still not fully understood. Studies conducted in this area of “target identification” are often rather complicated, and frequently comparable with the literal search for the needle in the haystack. Here, a combination of two distinct model organisms, the nematode *Steinernema feltiae* and the budding yeast *Saccharomyces cerevisiae*, seems to be a good choice to investigate the activity and possible modes of action of such redox modulating compounds upfront, *i.e.*, before more extravagant studies are planned and conducted. In the case of *Steinernema feltiae*, a whole multicellular organism is available rather readily for studies and can be employed among others to study uptake and distribution processes. *Steinernema feltiae* is a “beneficial” nematode used extensively by gardeners against agricultural pests, such as the larvae of the crane fly. The nematode itself is easy to obtain, culture and handle and can be used as a robust organism for basic toxicity screens. Using yeast, it is possible to combine the molecular biological “chemogenetic screening” on the one hand, and the various semi-quantitative (dye- and/or antibody-based) fluorescent staining and subsequent scoring techniques of the “intracellular diagnostics” on the other to obtain a first insight into the various biochemical events triggered or controlled by specific redox modulating agents. Nonetheless, both systems are clearly not “mammalian” and the limitations of such a platform based on organisms with a distinctively different metabolism compared to higher animals and humans always need to be born in mind.

## 2. Results and Discussion

### 2.1. Pre-Screen for Toxicity in Two Selected Model Organisms

The first task of this study has been the selection of relevant model systems. As human cells are rather difficult (and expensive) to culture, a more simple, easy-to-use model organism is required for a general pre-screening of activity in order to identify the most likely candidates for high activity and to assign a low priority to any entirely inactive agents. Ideally, such a simple system suitable for pre-screening should also be useful for subsequent intracellular diagnostics. Here, yeast first comes to mind. Indeed, yeast has already been employed successfully as a model system to study the toxicity of inorganic selenium compounds, such as SeO_3_^2−^ and SeO_4_^2−^. Since the response of yeast cells to these two selenium salts is well-documented, both have been used here for comparison [9,10,11,12]. As yeast is a single cell organism, however, another, also readily available organism has been used as a “control”, namely the nematode *Steinernema feltiae*. The latter represents an agriculturally relevant nematode which is not pathogenic, is easy to cultivate and generally provides reproducible and reliable results in an intact, multicellular living organism [13,14].

**Figure 2:**
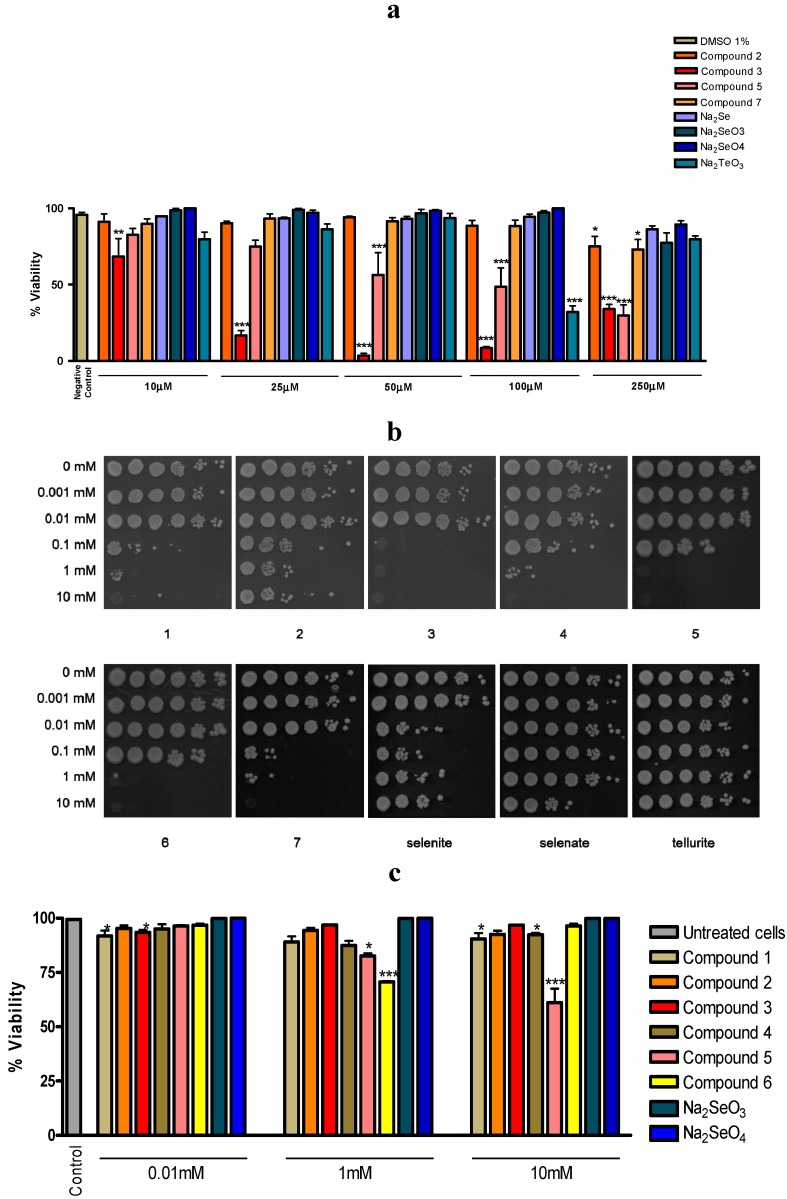
Most selenium and tellurium compounds investigated exhibit a pronounced toxicity established in two independent pre-screens based on a nematode and yeast. **Panel a:** Toxicity against the agricultural nematode *Steinernema feltiae*; **Panel b:** Toxicity against the *Saccharomyces cerevisiae* strain BY4741 using the spot test. Cells were treated with increasing concentrations of compounds (0.1 mM to 10 mM) for 3 h. After treatment, they were collected by centrifugation, washed with, and resuspended in, physiological saline, diluted, and spotted onto YPD plates to determine cell survival; **Panel c:** Quantification of dead *Saccharomyces cerevisiae* strain BY4741 cells by staining with PI and flow cytometry. Cells were treated with increasing concentrations of compounds, collected by centrifugation, washed with, and resuspended in, physiological saline. They were then stained with PI and stained, dead cells were quantified by flow cytometry. The values represent means with SD error bars from two separate experiments, and statistical significances have been calculated using a one-way ANOVA followed by Bonferroni’s multiple comparison test or Kruskal-Wallis followed by Dunn’s multiple comparison test. * denotes values significantly different from the control. * *p* < 0.05; ** *p* < 0.01; *** *p* < 0.001. See text for experimental details.

Figure 2 shows the impact of selected selenium and tellurium compounds on the survival of *Steinernema feltiae* (Figure 2a) and *Saccharomyces cerevisiae* (Figure 2b,c). In the case of yeast, two independent assays have been employed, one using an agar spotting method (Figure 2b), the other a cell survival assay based on propidium iodide (PI) staining and counting of dead cells immediately after treatment with the compounds (Figure 2c). Some of the substances tested seem to affect both, the survival of the nematodes and the growth of yeast, such as compound **5** (and to some extent compound **3**), whilst the others (such as compounds **6** and **7**) are only toxic against either yeast or nematodes. Interestingly, these results identify compound **5** as being particularly active. In the past, compound **5** and structurally closely related tellurium compounds have been studied extensively in human cancer cell lines and there have been identified as being amongst the most powerful, redox-modulating cytotoxic chalcogen agents available so far [3,15]. The results obtained in our simple nematode- and yeast-based assays therefore confirm this pronounced toxicity and also support the idea that such simple assays may be useful in pre-screening and subsequently pre-selecting compounds.

Nonetheless, there are exceptions which caution against the orthodox use of just one assay system, especially yeast. It seems that yeast is particularly sensitive to certain selenium compounds. Whilst the results obtained for the selenium compound **3** are comparable in yeast, *Steinernema feltiae* and to some extent even in human macrophage cell culture, other compounds, such as selenite, seem to affect yeast but not so much *Steinernema feltiae* (Figure 2a,b) [3]. Hence, whilst most compounds show a similar toxicity in the nematode and yeast assay, which more or less corresponds to their cytotoxicity in human cell lines, and whilst SeO_3_^2−^ is clearly a special case with a particular chemical reactivity (see below), there can be notable exceptions. It is therefore advisable to use at least two pre-screens for biological activity in tandem, such as a combination of nematodes and yeast, *i.e.*, a combination which seems to cover a wider range of activities, just to ensure that particular, perhaps also exceptional activities, do not escape our attention. Nonetheless, the inherent limitations still associated with employing a non-mammalian combination of organisms must be kept in mind. It is possible, for instance, that there are distinct differences in the uptake, metabolic pathways and excretion for selenium and tellurium compounds in yeast, worms and mammalian systems, and that such differences may form the basis for an exceptional toxicity of selenium compounds in yeast. It is also possible that yeast is not exceptionally sensitive to selenium compounds, but rather resilient against poisoning by tellurium compounds. Indeed, there are various tellurium-tolerant fungi which incorporate tellurium into sulfur-containing amino acids to form tellurocysteine and telluromethionine [16]. In any case, once a more general activity has been detected, it is certainly necessary to study the relevant compounds in more sophisticated test systems and in considerably more detail. This, of course, will ultimately also include the use of mammalian and eventually human model systems.

### 2.2. From “Activity” to Mechanistic Considerations: Microscopy, Chemogenetic Screening and “Intracellular Diagnostics”

The results obtained in *Steinernema feltiae* and the *Saccharomyces cerevisiae* BY4741 strain together have confirmed a significant toxicity of some of the selenium and tellurium compounds, such as compounds **3**, **5**, **6** and **7**, which is reflected in human cell cultures [2,3]. These assays seem to be able to distinguish between certain compounds that are active and hence interesting, and others which are not. Nonetheless, such a pre-selection tool mainly addresses the question if compounds are eventually active, and not necessarily why they are active.

**Figure 3:**
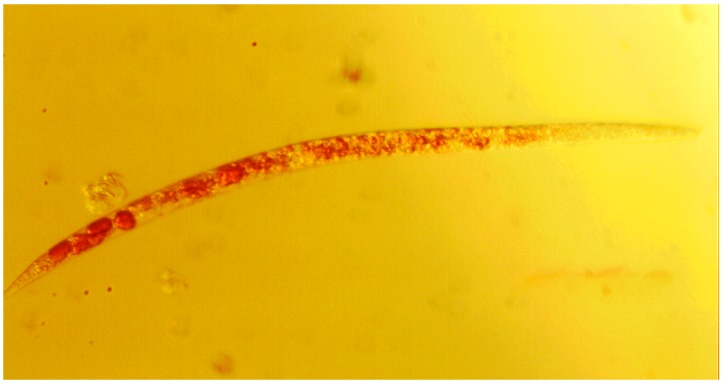
Microscopic image of a representative *Steinernema feltiae* nematode showing the enrichment of compound **5** (red color inside the nematode). 10-fold magnification on a TR 200 VWR International microscope.

The next step therefore needs to consider the possible and/or likely causes for such toxicity. Here, both model organisms offer certain additional insights into the effects of such compounds on the living organism. To begin with, the nematode is an ideal organism to study under the light microscope at rather modest magnification (e.g., 10-fold). It is a comparably simple organism and transparent, which enables us to study the distribution of (colored) compounds inside the nematode. As Figure 3 illustrates, compound **5** has apparently entered the nematode and has become enriched inside. This is a clear indication that the compound has been taken up and is distributed across the organism. Besides this simple but relevant “uptake” and “distribution” experiment, more detailed studies are also possible using nematodes such as *Steinernema feltiae* or *Caenorhabditis elegans*. The precise distribution of compounds can be studied, the organs and types of cells affected inside the nematode can be determined (e.g., cuticle, digestive tract) and such nematodes can even be stained and monitored under the fluorescence microscope.

Nonetheless, the nematode-based whole organism model cannot provide mechanistic information on the cellular level. Here, the yeast system offers a powerful alternative. As all of the compounds used in this study are known to be redox active based on structural considerations, *in vitro* redox assays and electrochemical investigations, a “redox link” is likely. To study this particular link, yeast endows the researcher with a set of tools which can be employed alone or in combination to address the “why” question in a eukaryotic organism. Here, yeast is particularly suited to uncover redox-related mechanisms, as redox-protecting systems are well conserved between yeast and mammals. Human cells, for instance, feature one additional (extracellular) superoxide dismutase to the cytosolic and mitochondrial one also existing in yeast. A similar situation is found in the case of catalase, where human cells possess one catalase enzyme. Nevertheless, additional ROS detoxifying components (such as peroxidases) may differ between mammals and yeast, and metabolic activity providing reducing equivalents for redox-maintenance generally is higher in yeast than in mammalian cell cultures. Still, many results that are obtained in the yeast system can be transferred to mammals with minor contingencies.

At the same time, yeast lends itself to extensive chemogenetic screening. As part of this approach, various yeast mutants lacking specific proteins and enzymes are employed and the toxicity observed for these mutants is then compared to the toxicity recorded in the corresponding wild type. Mutants that are more sensitive (or resistant) to a given compound when compared to the wild type may hint towards a possible link between the particular protein/enzyme lacking in the mutant and its role in metabolism or defense, and the compound in question. As knock-out mutants in all non-essential genes can be generated in yeast easily, or can be obtained from commercial suppliers such as EUROSCARF, chemogenetic screening is comparably easy to perform. This approach in some aspects also mirrors investigations concerned with the development of drug/pesticide resistance, where the resistance to a given compound provides hints of the pathways involved based on the overexpression of certain proteins. In both cases, such hints may then be used to guide further investigations by an array of cell-based analytical techniques we have branded as “intracellular diagnostics” [17].

**Figure 4:**
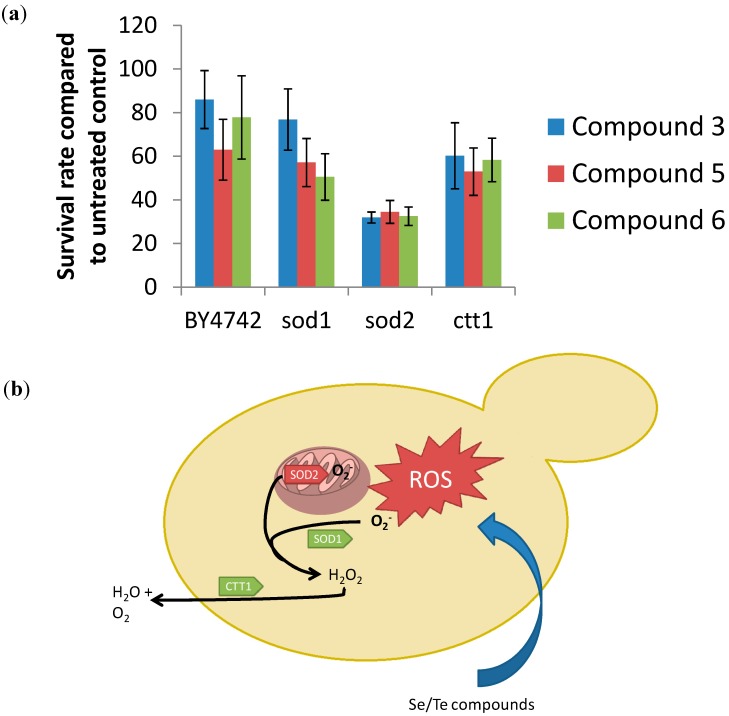
Chemogenetic screening of selected compounds using BY4742 wild type and the corresponding mutant strains of *Saccharomyces cerevisiae*. This screen indicates that the mutant deficient in the antioxidant enzyme Sod2 is particularly sensitive to the most active chalcogen compounds studied. Whilst no firm conclusions can be drawn, a redox link between the compounds on one side, and mitochondrial superoxide/superoxide dismutase and cell death on the other is likely. See text for experimental details. **Panel a:** Chemogenetic screening of yeast mutants impaired in ROS-detoxification in the BY4742 background compared with the BY4742-wild type. The results suggest an enhanced susceptibility of a *Δsod2* mutant against compounds 3, 5 and 6; **Panel b:** Scheme illustrating the mode of action of selenium- and tellurium containing compounds tested. The compounds seem to trigger the formation of superoxide in the mitochondria, since a mutant of a mitochondrial superoxide-dismutase is hypersusceptible to the tested compounds (indicated in red) mutants of the cytosolic superoxide-dismutase and catalase show no difference to the wild type regarding the resistance to the tested compounds.

Indeed, we have recently shown that such a basic chemogenetic screen in yeast focusing on mutants that lack one (or several) antioxidant enzymes is rather revealing [3]. Besides the wild type strain BY4742, mutants deficient in Sod1 (superoxide dismutase 1), Sod2 and Ctt1 (cytosolic catalase) have been considered. As Figure 4 illustrates, a particularly high toxicity of compounds **3**, **5** and **6** against the *sod2* mutant strain can be observed. This strain shows a survival rate of just 40% for these compounds, whilst under the same conditions, the other “redox impaired” mutants, such as the *sod1* or the *ctt1* mutant, show revival rates of 60% and higher. Such differences support the notion of a distinct redox link and also provide a strong hint that mitochondrially-based Sod2 and/or its substrate, namely O_2_^•−^, are somehow involved in the biological action of these selenium and tellurium compounds.

To investigate this redox hypothesis further, we have therefore employed a range of selective fluorescent dyes which belong to the emerging toolkit of intracellular diagnostics and, as these dyes do not lyse or kill the cell, can be used to monitor intracellular events even in a time resolved manner. Out of the arsenal of dyes available to date, we have evidently selected a range of “redox indicators” to begin with (in the following such diagnostic dyes are highlighted in bold) [4,18,19].

**Figure 5:**
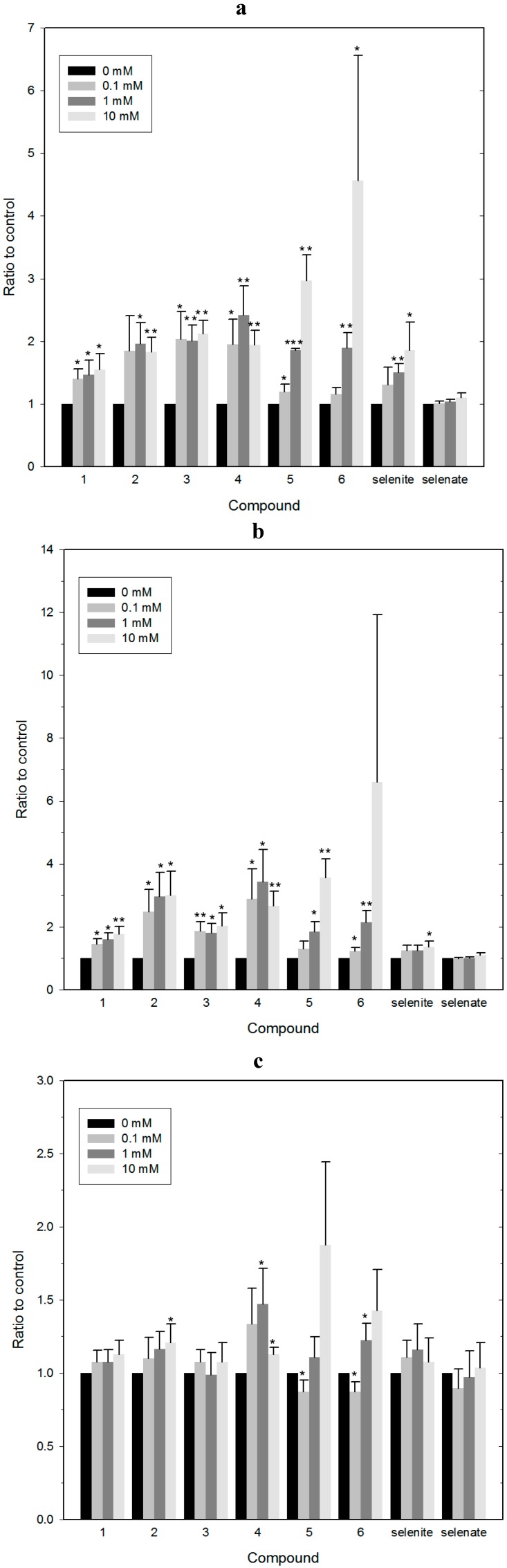
Estimating intracellular redox changes by employing selective, redox sensitive fluorescent dyes. **Panel a:** Production of ROS. Cells were treated with increasing concentrations of selected selenium and tellurium compounds and the total ROS production after treatment was estimated by staining with DCFDA; **Panel b:** Production of O_2_^•−^. Cells were treated with increasing concentrations of compounds and the level of mitochondrial O_2_^•−^ was estimated using MitoSOX Red as mitochondrial O_2_^•−^ indicator;. **Panel c:** Production of ^1^O_2_. Cells were treated with increasing concentrations of compounds and intracellular levels of ^1^O_2_ were assessed using the Singlet Oxygen Sensor Green reagent. The values represent medians with SD error bars from at least four separate experiments. Statistical significances have been calculated using a one sample Student’s *t* test. * denotes values significantly different from the control. * *p* < 0.05; ** *p* < 0.01; *** *p* < 0.001.

As the chemogenetic screen shown in Figure 4 has already pointed towards the involvement of ROS, namely in form of O_2_^•−^, and since raised concentrations of oxidative stressors could indeed cause non-tolerable intracellular redox imbalances and cell death, we have therefore measured the total ROS levels in BY4741 yeast cells treated with the synthetic Se- and Te-containing compounds first, using 2',7'-dichlorodihydrofluorescein diacetate (DCFDA) as an indicator dye and the two inorganic selenium salts as benchmark. As shown in Figure 5a, synthetic Se- and Te-containing organic redox modulators generate high levels of ROS, in some instances already at compound concentrations of 100 µM. Whilst these results are rather revealing, it should be emphasized that the use of DCFDA as a general stain for ROS provides initial information regarding the redox state of the cell (primarily its cytosol), yet the use of this indicator dye is not entirely unproblematic. DCFDA is hydrolyzed by certain esterases as it enters the cell and the intracellular concentration of its oxidized, fluorescent form therefore also depends on esterase activity—and not only on the concentration of ROS. In addition, DCFDA reacts with some ROS (mainly peroxides, H_2_O_2_), but not with all ROS and in any case not with the same kinetics. This may impact on the ability of DCFDA to monitor ROS levels in the cell adequately and in a differentiated manner.

Interestingly, there is a notable absence of any major difference between Se- and Te-containing redox modulators. Furthermore, SeO_3_^2−^ and SeO_4_^2−^ do not seem to induce raised ROS levels. It therefore appears that the quinone moiety is mostly responsible for the increase in ROS concentrations observed, whilst the presence of either selenium or tellurium has only a minor impact. This finding is hardly surprising, as the quinone moiety is well known to undergo redox cycling in the presence of O_2_ (hence generating O_2_^•−^) and has been “added” to the chalcogen moiety intentionally to act as a ROS generator [20,21].

To return to our findings from the chemogenetic screen and hence to gain a deeper insight into which particular ROS may be involved in the toxicity of redox modulators in yeast, we have employed a selective assay for mitochondrial O_2_^•−^ based on the fluorescent indicator dye MitoSOX Red. Figure 5b indicates that all synthetic Se- and Te-containing compounds tested also raise intracellular levels of mitochondrial O_2_^•−^.

Indeed, the general trend observed in Figure 5a using the DCFDA stain can also be observed in Figure 5b, with increases in the respective oxidant concentrations of around two- to three-fold. This indicates that most of the ROS formation observed is probably due to an (initial) formation of O_2_^•−^. As before, quinone-containing compounds seem to be particularly active, whilst the inorganic salts (SeO_3_^2−^ and SeO_4_^2−^) are not.

Besides O_2_^•−^, singlet dioxygen ^1^O_2_ is also sometimes formed in cells upon stimulation with redox agents and may represent another ROS type involved in the activity of the compounds under investigation. Indeed, there are several reasons to consider the presence of ^1^O_2_ as part of this study. Firstly, most of the compounds used are colored and hence may exert a photosensitizing activity. Secondly, SeO_3_^2−^ is known to cause DNA double-strand breaks (DSBs), and its chemistry is associated with the most potent types of ROS, such as the hydroxyl radical (^•^OH) and ^1^O_2_ (see below). And thirdly, ^1^O_2_ represents a branch of ROS formation which runs independently of the mitochondrial O_2_^•−^ cascade or the ROS production by NADPH oxidases. As Figure 5c indicates, ^1^O_2_ levels, measured with the fluorescent dye Singlet Oxygen Sensor Green, did not increase considerably in the presence of the compounds. Only small, up to 1.5-fold increases were observed for compounds such as **4**, and only at high compound concentrations of 10 mM, which are hardly relevant for potential biological applications. SeO_3_^2−^ showed a very weak increase as well, rejecting the hypothesis that this particular compound causes DNA damage via the formation of high amounts of ^1^O_2_.

**Figure 6:**
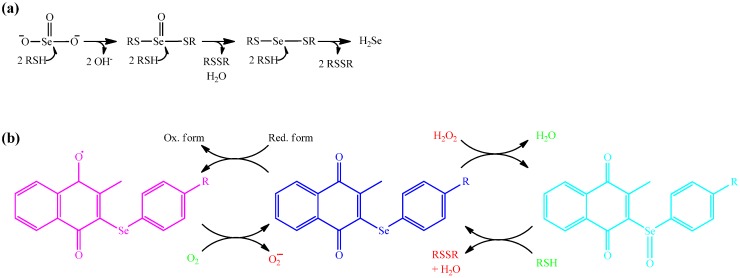
Schematic overview of the chemistry and reactivity of selected selenium and tellurium compounds under physiological conditions, *i.e.*, in the presence of reducing thiols. **Panel a:** SeO_3_^2−^ reacts mostly spontaneously and rapidly with up to six thiol equivalents (including GSH and protein thiols) to generate first an unusual selenotrisulfide and ultimately a mixed selenodisulfide (selenylsulfide) and hydrogen selenide (H_2_Se). In contrast, neither SeO_4_^2−^ nor the organic selenium and tellurium compounds studied seem to exhibit this particular kind of chemistry; **Panel b:** Most of the organic compounds tested rather generate O_2_^•−^ (probably due to the presence of a naphthoquinone moiety) or facilitate the reaction of existing ROS with protein thiols. Whilst this simple illustration cannot explain the entire biochemistry behind such compounds, it provides some hints why SeO_3_^2−^ and the organochalcogens tested are sometimes equally toxic, yet SeO_3_^2−^ reacts rather differently compared to most of the other compounds and, notably, also induces severe damage to DNA (and hence may not be the best choice of trace element supplement).

Hence, it appears that the selenium and tellurium compounds investigated in yeast predominantly generate (mitochondrial) O_2_^•−^ compared to other ROS, a finding which agrees well with our chemogenetic screen and recent studies conducted in mammalian cells. This notion is also in line with the particular “chemistry” of such quinone-containing agents which is illustrated in Figure 6. If O_2_^•−^ indeed is the ROS ultimately responsible for cellular damage (see below) and cell death, or if it is converted to H_2_O_2_ and to the ^•^OH radical—which in turn cause the damage and cell death observed—remains to be shown (see discussion below). Similarly, whilst we propose that there is a direct causal relationship between ROS production, cellular damage and cell death in case of many selenium and tellurium compounds, including inorganic selenium salts, one may speculate that such compounds themselves are fairly unreactive, yet may become metabolically converted to a common intermediate which subsequently triggers the formation of ROS. Such a metabolic conversion may be independent of the type and concentration of compound applied. Alternatively, these compounds may not generate ROS by themselves but interfere with either ROS-generating or ROS-detoxifying enzymes. They may, for instance, cause some hitherto unnoticed damage to the cell or to a particular cellular compartment (such as the cytoskeleton, the mitochondria, the endoplasmic reticulum), which would then result in an oxidative burst. Then again, such compounds may not generate ROS at all, either directly or indirectly, but may inhibit a ROS detoxifying system, such as a particular reductase, instead. Indeed, some recent studies have identified the human enzyme thioredoxin reductase as a potential target for selenium and especially also for tellurium compounds [22,23,24].

In any case, a significant increase in intracellular concentrations of ROS upon application of certain selenium and tellurium compounds seems to occur consistently across different organisms. This raises the question which kind of damage such ROS subsequently cause inside the cell. Here, membranes, redox sensitive proteins of the cellular thiolstat and DNA represent prominent targets. As the effect on membranes and on proteins has been studied rather extensively before [25,26,27,28,29], we have explored here the effects on DNA using an assay indicative of DSBs.

### 2.3. DNA as Target: The Special Role of SeO_3_^2−^

**Figure 7:**
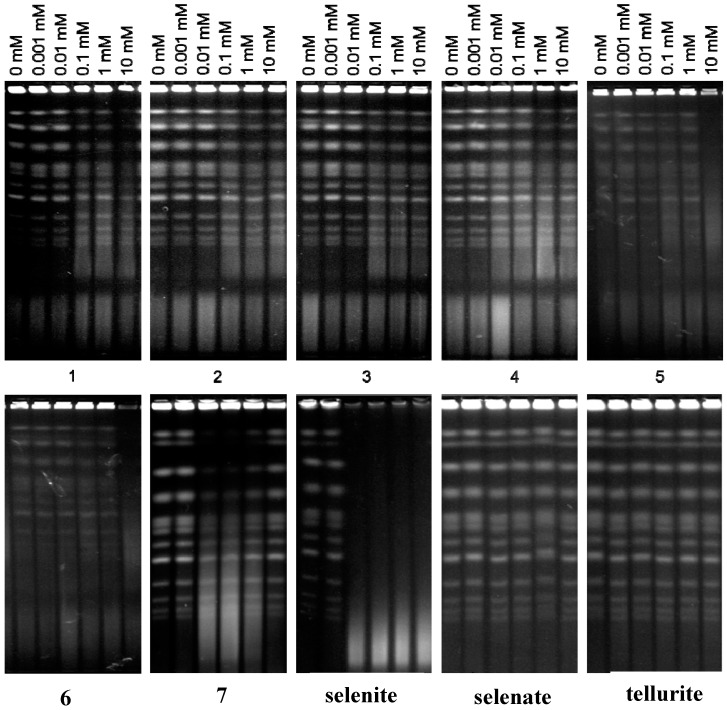
Induction of DSBs upon treatment with increasing concentrations of selenium and tellurium compounds. For analysis of DSBs, treated cells were embedded into low melting-point agarose to generate plugs, in which the cells were lysed. After lysis, the plugs were loaded into wells of the gel and chromosomal DNA was subjected to electrophoretic separation. After electrophoresis, the gels were stained with ethidium bromide, visualized using a UV transilluminator and photographed. In all cases, experiments were performed in triplicate and representative gels are shown.

Increased ROS levels are often capable of producing DSBs, and this particular damage may well also explain the notable toxicity associated with such agents [30]. A possible induction of DSBs by the various selenium and tellurium compounds has therefore been investigated, using data for the inorganic selenium and tellurium salts for comparison. In line with our previous reports [9,10,11], SeO_3_^2−^ induces DSBs in yeast in a dose-dependent manner (Figure 7). In stark contrast, neither SeO_4_^2−^ nor any of the selenium or tellurium compounds tested (including TeO_3_^2−^) cause any significant DSBs. As there is no significant difference between the most and the least toxic compounds in this assay, it is unlikely that DSBs form a decisive part of the intracellular mechanism(s) underlying the rather pronounced toxicity observed in this study for some of the compounds, such as compounds **3**, **5**, **6** and **7**.

One should note, however, that among all of the compounds tested in this study, it is precisely SeO_3_^2−^ which is used by people in practice. Here, selenite increasingly represents the selenium component of commercial trace element supplements (instead of selenomethionine which can be enriched in yeast cultivated in an appropriate source of selenium). As such nutritional supplements are freely available in supermarkets and their use is not controlled, considerable care should be taken. Whilst it is unlikely that SeO_3_^2−^ reaches cellular DNA easily, it still has the potential to cause DSBs and hence also mutations which in the long term may result in the formation of cancer [11].

The fact that SeO_3_^2−^ seems to be fairly potent in causing or sustaining DNA damage, whilst many selenium and tellurium compounds—despite the fact that they up-regulate intracellular concentrations of ROS—do not, also deserves some comment. Selenite is a highly reactive inorganic form of selenium which reacts spontaneously—and rather rapidly—with many biological thiols, including GSH and numerous cysteine-containing proteins. This reaction is sketched out in Figure 6a. It is often assisted by specific enzymes and results in some rather unusual chemical structures, and may ultimately end in the formation of disulfides, selenodisulfides (selenylsulfides), selenotrisulfides and H_2_Se. 

Whilst a direct “chemical attack” by SeO_3_^2−^ on DNA is unlikely from a purely chemical point of view, SeO_3_^2−^ is well posed to inhibit many (cysteine-containing) enzymes, including proteins and enzymes involved in DNA repair, and hence may increase the extent of DSBs in a more indirect manner, e.g., by inhibiting homologous recombination (HR). In fact, HR is one of the two main DSB repair mechanisms that has been shown previously to play a crucial role in repairing DSBs induced by SeO_3_^2−^ [10]. Here, one should notice that DSBs usually are not generated directly. Other types of damage may result in DSBs via DNA replication, attempted and aborted DNA repair, and by co-existence of adjacent single-strand breaks in opposite DNA strands.

It is doubtful that the Se- and Te-compounds investigated as part of this study can undergo the same kind of chemistry associated with SeO_3_^2−^. Consequently, a mechanism other than induction of DSBs seems to be responsible for the pronounced toxicity of synthetic Se- and Te-containing organic redox modulators used in the present study (see also Figure 6b). Still, we have been able to demonstrate that those compounds generate considerable amounts of ROS, primarily O_2_^•−^, and one may well speculate that such ROS can also induce or sustain DNA damage. It is therefore noteworthy that we could not find any DSBs despite an apparent increase in ROS levels.

In the future, it would therefore be worthwhile to consider other potential intracellular targets for the compounds investigated as part of study. Besides an increase in ROS concentrations, which may indirectly damage numerous biomolecules, a direct interaction with cysteine proteins of the thiolstat is likely. At the same time, considerable care should be taken with the uncontrolled public release and consumption of selenite, as SeO_3_^2−^ in a biological context seems to be a rather potent and aggressive selenium species to deal with.

## 3. Experimental Section

### 3.1. Yeast Strain and Media

The *Saccharomyces cerevisiae* strains used in this study were BY4741 (*MATa his3Δ1 leu2Δ0 met15Δ0 ura3Δ0*) and BY4742 (as BY4741 with *MATα*) from EUROSCARF. The composition of the media was the same as described previously [9,10,11,12].

### 3.2. Chemicals

Sodium selenite (Na_2_SeO_3_) was obtained from Merck KGaA (Darmstadt, Germany). Sodium selenate (Na_2_SeO_4_) and sodium selenide (Na_2_Se) were purchased from Sigma-Aldrich Chemie GmbH, (Steinheim, Germany). Sodium tellurite (Na_2_TeO_3_) and sodium tellurate (Na_2_TeO_4_) were obtained from Alfa Aesar (Karlsruhe, Germany). Compounds 1-7 were synthesized according to literature [2]. The chemical structures of all compounds tested in this study are shown in Figure 1b.

### 3.3. Nematode Assay Based on Steinernema Feltiae

*Steinernema feltiae* has been obtained from Sauter and Stepper (Ammerbruch, Germany). The assay has been performed as described in the literature [31]. Results are expressed as mean ± standard deviation (SD) and the statistical significance has been determined by one-way ANOVA followed by Bonferroni’s multiple comparison test. A value of *p* ≤ 0.05 is considered statistically significant.

### 3.4. Growth and Treatment Conditions for the Yeast Assay

Yeast was cultured and treatments were carried out in accordance with our previous studies [9,10,11]. Briefly, yeast cells were grown in YPD medium overnight. The overnight culture was then used to inoculate fresh YPD. Incubation of fresh YPD culture continued until the cell suspension reached a density of 2 × 10^7^ cells/mL. Cells were then treated with increasing concentrations of compounds at 30 °C for 3 h with shaking. After treatment, cells were collected by centrifugation, washed twice with, and resuspended in, physiological saline (0.9% NaCl), diluted, and spot plated onto YPD plates to determine cell survival and any potential cytotoxic effects. In addition, to identify dead cells immediately after the treatment, propidium iodide staining (7.5 μL of 1 mg/mL solution; Sigma-Aldrich Chemie GmbH) for 5 min and subsequent analysis with a BD FACS Canto II flow cytometer (BD Biosciences, Franklin Lakes, NJ, USA) in the PE channel was employed to quantify dead cells. The control (untreated) sample was set at 100% of viability in each individual experiment. Final data represents the average of two independent experiments. Results are expressed as mean ± standard deviation (SD) and the statistical significance has been determined by Kruskal-Wallis followed by Dunn’s multiple comparison test. A value of *p* ≤ 0.05 is considered statistically significant.

Yeast strains that were used for chemogenetic screening are derivatives of the BY4742 wild type background. To investigate the role of ROS for the toxicity of the compounds, BY4742-derived mutants for the cytosolic superoxide dismutase *sod1* (YJR104c), mitochondrial superoxide-dismutase *sod2* (YHR008c) and the cytosolic catalase *ctt1* (YGR088c) were used. Extensive details of the relevant experimental procedures have been published by us recently [3]. In brief, yeast cells were grown overnight in complete synthetic dropout medium (CSM, 7 g/L yeast nitrogen base, for medium, (Hunstaton, Norfolk, UK); 0.8 g complete dropout, (Bio101, Vista, CA, USA), 40 g/L glucose (Carl Roth, Karlsruhe, Germany)) at 28 °C and 210 rpm and adjusted to an OD_600_ = 1. Compounds (dissolved in DMSO) were added and the culture was cultivated further for 16 h. Subsequently, yeast cultures were plated as serial dilution (1:1; 1:10^2^, 1:10^4^, 1:10^6^). For quantification, the numbers of colonies of the 1:10^6^ dilution were counted and the survival rate was calculated as quotient of the colony forming units (cfu) of the treated culture and the untreated control × 100. The values represent the mean with standard deviation of eight individual samples within one experiment. Statistical significances have been determined using Student’s *t*-test with * *p* < 0.05; ** *p* < 0.01; *** *p* < 0.001.

### 3.5. Pulsed-Field Gel Electrophoresis

Pulsed-field gel electrophoresis (PFGE) experiments were performed as described previously [9,10,11]. Briefly, treated and untreated cells were washed twice with, and resuspended in, 50 mM EDTA (pH 7.5) at a density of 6.25 × 10^8^ cells/mL. Thereafter, 80 μL of this suspension were mixed with 20 μL of a buffer composed of 2 M sorbitol, 1 M citrate, 0.5 M EDTA (pH 7.5) and 10% *β*-mercapto-ethanol (*β*-ME). Subsequently, 2.5 μL of lyticase (10 mg/mL; Sigma-Aldrich Chemie GmbH) and 100 μL of 1% low melting-point agarose (Sigma-Aldrich Chemie GmbH) in 0.125 M EDTA (pH 7.5) were added. The resulting cell suspension was first equilibrated at 45 °C and then transferred immediately into the plug moulds and cooled until it solidified. After removing them from the moulds, the plugs were incubated in a buffer consisting of 0.5 M EDTA, 0.4% *β*-ME and 0.01 M Tris-HCl (pH 8.0) for 2 h. They were then lysed at 37 °C in 0.5 M EDTA, 0.01 M Tris-HCl (pH 8.0), 1% *N*-lauroylsarcosine and 0.5 mg/mL proteinase K (Amresco, Solon, OH, USA) overnight. The next day, the plugs were incubated at 37 °C for 2 h in a buffer composed of 1 mM Pefabloc SC AEBSF (Roche Applied Science, Mannheim, Germany), 1 mM EDTA and 10 mM Tris-HCl (pH 8.0) and then rinsed twice with 50 mM EDTA (pH 7.5). The plugs were stored in a buffer consisting of 1 mM Pefabloc SC AEBSF and 0.5 M EDTA (pH 8.0) at 4 °C until used. Before loading them into the wells, the plugs were equilibrated twice in a buffer composed of 10 mM Tris-HCl and 1 mM EDTA (pH 7.5). Loaded wells were covered with a 1% agarose gel. Electrophoresis was performed in 1% agarose gel and 0.5 x TAE buffer (20 mM Tris acetate, 1 mM EDTA pH 8.0) using a CHEF MAPPER^®^ XA SYSTEM (Bio-Rad, Hercules, CA, USA) with a constant voltage of 4.5 V/cm for 23 h at 14 °C with a switch time of 60 to 120 s. After electrophoresis, the gels were stained with 1 μg/mL ethidium bromide for 2 h, destained overnight in TAE buffer containing RNase (2 μg/mL), visualized on a UV transilluminator and photographed with a GDS 7500 Gel Documentation System (UVP, Upland, CA, USA).

### 3.6. Detection of ROS

Treated and untreated cells were pelleted, washed twice with, and resuspended in 0.1 M potassium phosphate buffer (pH 7.4). To determine the overall intracellular ROS levels, cell cultures (1 × 10^6^ cells/mL) were incubated in the dark with 2',7'-dichlorodihydrofluorescein diacetate (DCFDA, Sigma-Aldrich Chemie GmbH) for 60 min at 30 °C in a final volume of 500 μL (the DCFDA working concentration was 10 μM). Furthermore, in order to gain a more specific insight into the concentrations of individual stressors, a 10 min incubation with 5 μM MitoSOX Red (MR) or 10 μM Singlet Oxygen Sensor Green (SOSG) (both from Invitrogen Ltd., Paisley, UK) was performed to assess the levels of (mitochondrial) superoxide radical anions (O_2_^•−^) and singlet dioxygen (^1^O_2_), respectively. Stock solutions of these fluorescent indicators were prepared according to the manufacturer’s instructions. After staining, cells were washed with, and resuspended in, ice-cold phosphate buffer. ROS levels were then estimated with a BD FACS Canto II flow cytometer (BD Biosciences) using a 530/30 nm FITC filter (for DCFDA and SOSG fluorescence) or a 585/40 nm PE filter (for MR fluorescence). 20,000 events were scored and data analysis was performed with the BD FACSDiva software. For each concentration, the ratio of sample to control fluorescence was calculated. Statistical significances of four individual experiments have been assessed using one sample Student’s *t*-test [32].

## 4. Conclusions

**Figure 8:**
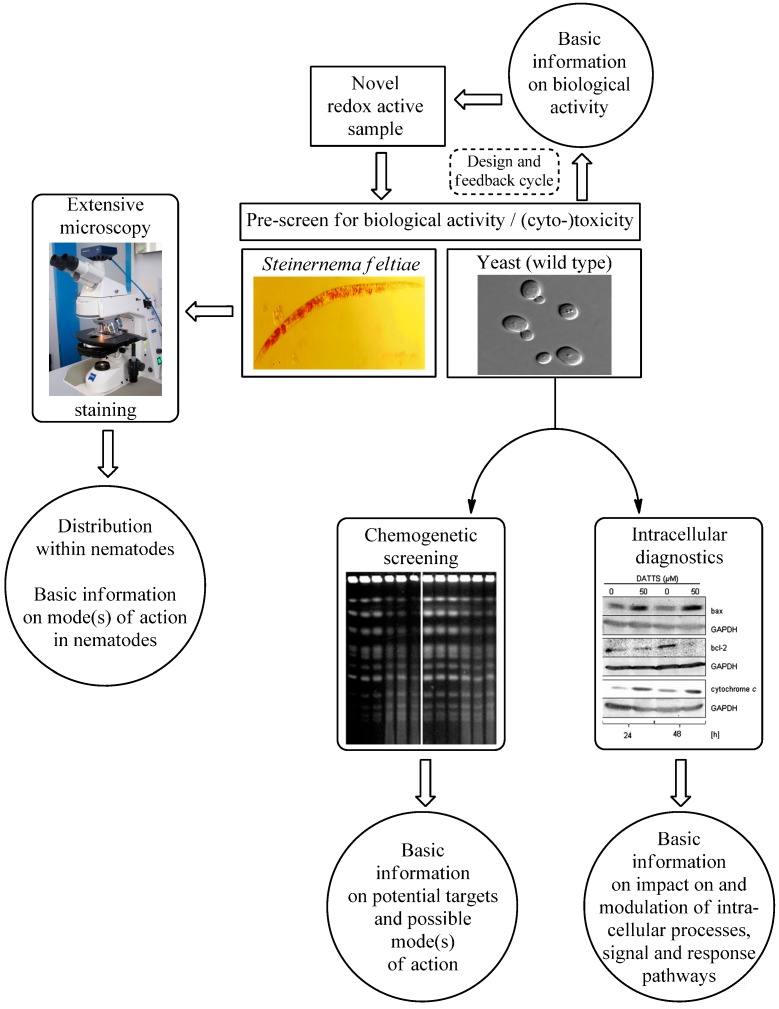
Schematic overview of the methodology employed to establish a basic insight into the biological activity and the likely mode(s) of action of (novel) redox active agents. The combination of the nematode and yeast assay enables a fast and efficient screen of novel compounds, including redox active plant products and secondary metabolites. Both model systems need to be considered in tandem to limit the number of organism-specific activities (e.g., selenium in yeast). In both cases, additional information can be obtained, such as uptake and distribution of compounds in the nematode or mechanistic information in yeast employing chemogenetic screening in conjunction with intracellular diagnostics.

Taken together, our studies have shown that it is possible to establish a robust and fairly straightforward pre-screen for redox active natural and synthetic compounds using a combination of a nematode and yeast assay (Figure 8). Both model organisms complement each other, and besides providing a first glimpse of the suspected biological activity of such compounds, they also enable more in-depth studies. Here, the nematode is particularly useful to study the uptake and distribution of a given compound in a small, primitive multicellular organism. Yeast, on the other side, enables researchers to go “deeper”, right down to the mode(s) of action by using a combination of chemogenetic screening and fluorescent dye based intracellular diagnostics. Needless to say, both models are necessarily imperfect, provide scope for discrepancies, and ultimately cannot substitute for more detailed studies in human cell culture and animals. The design and biochemistry of a human cell, for instance, differs from the one of a nematode cell or yeast. There are significant metabolic differences at various levels, from the single cell to the whole organism. Nonetheless, redox-protecting systems are well conserved between yeast and mammals and the results that are obtained in the yeast system, for instance, can be transferred to mammals with minor contingencies. A combination of several simple organisms therefore is still able to provide considerable impetus and direction for more complex and time consuming studies in more advanced systems. In the specific context of this particular study, such “simple” studies may also provide early warnings of potentially dangerous agents, such as SeO_3_^2−^.
